# Wharton County Medical Society

**Published:** 1903-10

**Authors:** J. M. Andrews

**Affiliations:** Secretary; Wharton, Texas


### Wharton County Medical Society.

This society was organized at Wharton, August 18, 1903, by Dr. Walter Shropshire, of Yoakum, Councilor for the DeWitt District. The following officers were elected: President, Dr. G. L. Davidson, Wharton; Vice-President, Dr. M. M. Pool, El Campo; Secretary, Dr. J. M. Andrews, Wharton; Treasurer, Dr. D. P. Redwine, El Campo; Board of Censors, Drs. W. D. Ray, East Barnard, J. W. Teague and S. M. Applewhite, Wharton.

Our membership is now fourteen, with fair prospects of increase at next meeting.

J. M. Andrews, Secretary.

Wharton, Texas, October 8, 1903.

